# Targeting Src in endometriosis-associated ovarian cancer

**DOI:** 10.1038/oncsis.2016.54

**Published:** 2016-08-15

**Authors:** R Manek, E Pakzamir, P Mhawech-Fauceglia, T Pejovic, H Sowter, S A Gayther, K Lawrenson

**Affiliations:** 1 Department of Preventive Medicine, Keck School of Medicine, University of Southern California Norris Comprehensive Cancer Center, Los Angeles, CA, USA; 2 Departments of Medicine and Pathology, Keck School of Medicine, University of Southern California, Los Angeles, CA, USA; 3 Department of Obstetrics and Gynecology, Oregon Health and Science University, Portland, OR, USA; 4 Department of Biomedical Science and Public Health, University of Derby, Derby, UK

## Abstract

The *SRC* proto-oncogene is commonly overexpressed or activated during cancer development. Src family kinase inhibitors are approved for the treatment of certain leukemias, and are in clinical trials for the treatment of solid tumors. Src signaling is activated in endometriosis, a precursor of clear cell and endometrioid subtypes of epithelial ovarian cancers (OCs). We examined the expression of phosphorylated Src (Src-pY416) in 381 primary OC tissues. Thirty-six percent of OCs expressed Src-pY416. Src-pY416 expression was most common in endometriosis-associated OCs (EAOCs) (*P*=0.011), particularly in clear cell OCs where 58.5% of cases expressed Src-pY416. Src-pY416 expression was associated with shorter overall survival (log rank *P*=0.002). *In vitro* inhibition of Src signaling using 4-amino-5-(4-chlorophenyl)-7-(dimethylethyl)pyrazolo[3,4-*d*]pyrimidine (PP2) resulted in reduced anchorage-independent and -dependent growth, and in three-dimensional cell culture models PP2 disrupted aggregate formation in Src-pY416-positive but not in Src-pY416-negative cell lines. These data suggest that targeting active Src signaling could be a novel therapeutic opportunity for EAOCs, and support the further pre-clinical investigation of Src family kinase inhibitors for treating OCs expressing Src-pY416.

## Introduction

*SRC* was the first oncogene to be described over 40 years ago. Since then, dysregulation of *SRC* and the other eight SRC family kinase (SFK) members has been shown to be important in the development of many solid tumor types.^1^ In normal tissues, Src is typically found in an inactive form, but during cancer development Src can become overexpressed or activated by mutations that increase its enzymatic activity. Src is a serine/threonine kinase and, when active, transduces signaling cascades through the STAT3/MYC, MAPK and PI3K pathways. Consequently, overexpression or mutation of Src is associated with a variety of tumorigenic phenotypes including angiogenesis, proliferation, invasion, motility and chemoresistance.^1, 2^

It is estimated that over 21 000 women in the United States will be diagnosed with epithelial ovarian cancer (OC) in 2015.^3^ OCs are the eighth most common cancer among women in developing countries, but the fourth most common cause of cancer death. OCs are particularly lethal, with 5-year survival rates of ~45%.^3^ There are four main histological subtypes of invasive epithelial OC—high-grade serous, endometrioid, clear cell and mucinous —with each histotype characterized by specific somatic alterations and biomarker expression.^4^ While histologically distinct, clear cell and endometrioid OCs share some common molecular and epidemiological features. Both commonly exhibit mutation or loss of the *ARID1A* tumor suppressor gene;^5, 6^ and endometriosis represents a defined precursor for these subtypes but not for the most common subtype, high-grade serous OC.^7, 8, 9, 10, 11^ Thus, collectively clear cell and endometrioid OCs are referred to as ‘endometriosis-associated ovarian cancers' (EAOCs). It has been shown that Src signaling is common in mucinous OC,^12, 13^ but the importance of Src in other OC subtypes is currently unknown. We have previously shown that Src signaling is active in *in vitro* models of endometriosis, as well as in the eutopic endometrium of endometriosis patients.^14^ This led us to hypothesize that Src may also be activated in EAOCs. In the current study, we investigated this hypothesis by first evaluating expression of active Src in primary OCs representing the main histological subtypes, and then using *in vitro* models of different OC subtypes to establish the effects of inhibiting Src. Our results indicate that Src inhibition may represent a novel therapeutic target for EAOC.

## Results

### p-Src expression in primary OCs

**Figure 1:**
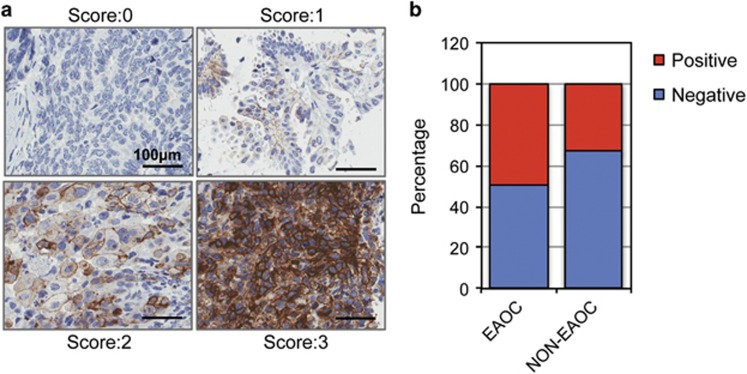
Expression of active Src (Src-pY416) in epithelial OC. (**a**) Representative images of Src staining in primary OCs. Staining was scored as negative (0), weak (1), moderate (2) or strong (3). (**b**) Forty-nine percent of all EAOCs express active Src.

We examined the expression of Src by immunohistochemical staining in 381 primary epithelial OC tissues representing the major histological subtypes, using an antibody that specifically recognizes the Src protein when phosphorylated at tyrosine 416 (Src-pY416) (Figure 1a) (Supplementary Table 1). This modification is associated with enhanced biological activity of Src and represents a surrogate marker of active Src signaling. The immunohistochemical staining data, stratified by clinico-histopathological characteristics, are summarized in Table 1. Thirty-six percent of OCs expressed Src-pY416. There were no significant associations between Src-pY416 positivity and clinical stage or tumor grade. However, we found a significant association between Src-pY416 expression and histological subtype, with Src-pY416 positivity strongly associated with EAOCs (*P*=0.012) but not with the serous subtype (*P*=0.139). Src-pY416 positivity was the most common in clear cell OCs, where 24/41 cases (58.5%) were positive. In this study, Src-pY416 was only detected in 4/21 mucinous OCs.

### p-Src expression is associated with worse patient outcomes

**Figure 2:**
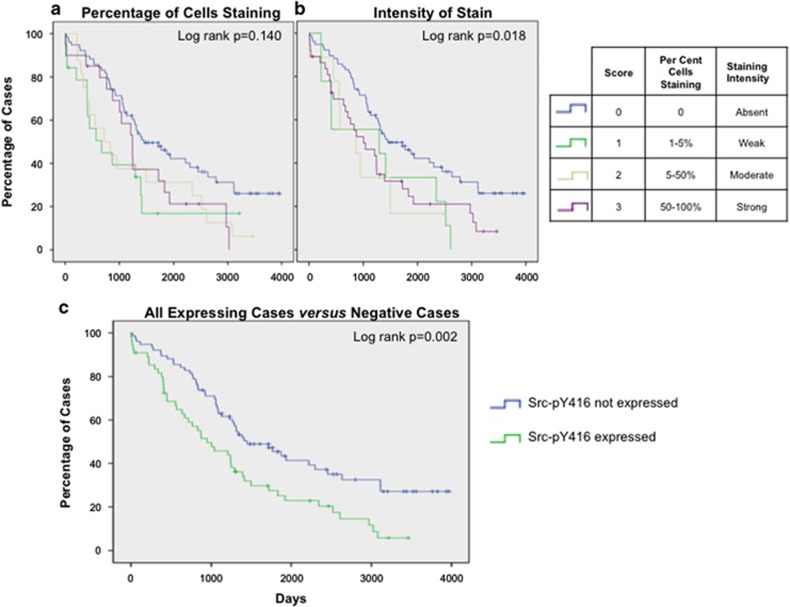
Patient outcome is associated with Src-pY416 expression. (**a**) Evaluating percentage of cells staining positive for active Src indicated a trend for poorer survival in Src-pY416 expressing tumors. (**b**) Staining intensity is associated with patient outcome, patients not expressing active Src have improved survival. (**c**) All expressing cases were compared with all negative cases; expression of active Src was associated with significantly shorter survival.

Survival data were available for 199/381 tumors. We tested for associations between active Src expression and survival using Kaplan–Meier survival analyses with log-rank testing. Samples with Src expression were first stratified into three groups based on the percentage or intensity of the staining. Src-pY416 expression was associated with worse overall survival based on staining intensity (log rank *P*=0.018), but was not significantly associated with survival based on the percentage of stained cells (log rank *P*=0.14), although the trend was the same (Figures 2a and b). When the results for staining intensity and percentage of positive cells were combined, the association with overall survival was stronger (log rank *P*=0.002) (Figure 2c). Median survival time was 498 days (16 months) less in tumors expressing Src compared with those that did not.

### p-Src expression in cell line models of OC histotypes

We have previously described the histology of 31 OC cell lines grown as two-dimensional (2D) monolayers and as three-dimensional (3D) spheroid cultures, and found that 3D cultures more closely mimic the histological differentiation and marker expression of human primary tumors than their 2D counterparts.^15^ However, the majority of OC cell lines show poorly differentiated histologies when cultured in 3D, despite having originated from differentiated tumor specimens.^15^ In the present study, we included additional 35 cell lines to identify other good models of the main OC histotypes. In this panel of cell lines, we found that only 28.6% (2/7) of serous cell lines resembled human serous OCs when grown in 3D, but 76.5% of clear cell OC cell lines (13/17) showed clear cell differentiation when grown as 3D spheroids. These results indicate that OV-177 and SHIN-3 are good models of serous OC, and that HCH-1, JHOC-5, JHOC-7, JHOC-9, KOC-7c, OV207, OVAS, OVISE, OVMANA, OVTOKO, RMG-II, SMOV-2 and TU-OC-1 are good models of clear cell OC. These results can be found in full in Supplementary Table 2.

We proceeded by evaluating Src-pY416 expression in epithelial OC cell lines grown in 2D and 3D. Of 64 OC cell lines evaluated, 34 OC (53%) exhibited Src-pY416 positivity in 2D and/or 3D cultures. Cell lines with strong Src-pY416 staining included Hey.A8, HEY-C2, TYK-nu, HAC-2, SMOV-2 and OVISE (of which the latter three are derived from clear cell tumors) (Supplementary Table 3). The remaining 30/64 (48.5%) cell lines did not express active Src. Src activity in 2D and 3D cultured cells was highly correlated (*R*^2^=0.52), indicating that Src signaling is not altered by culture conditions.

### Phenotypic consequences of Src-pY416 inhibition in *in vitro* models of OC

**Figure 3:**
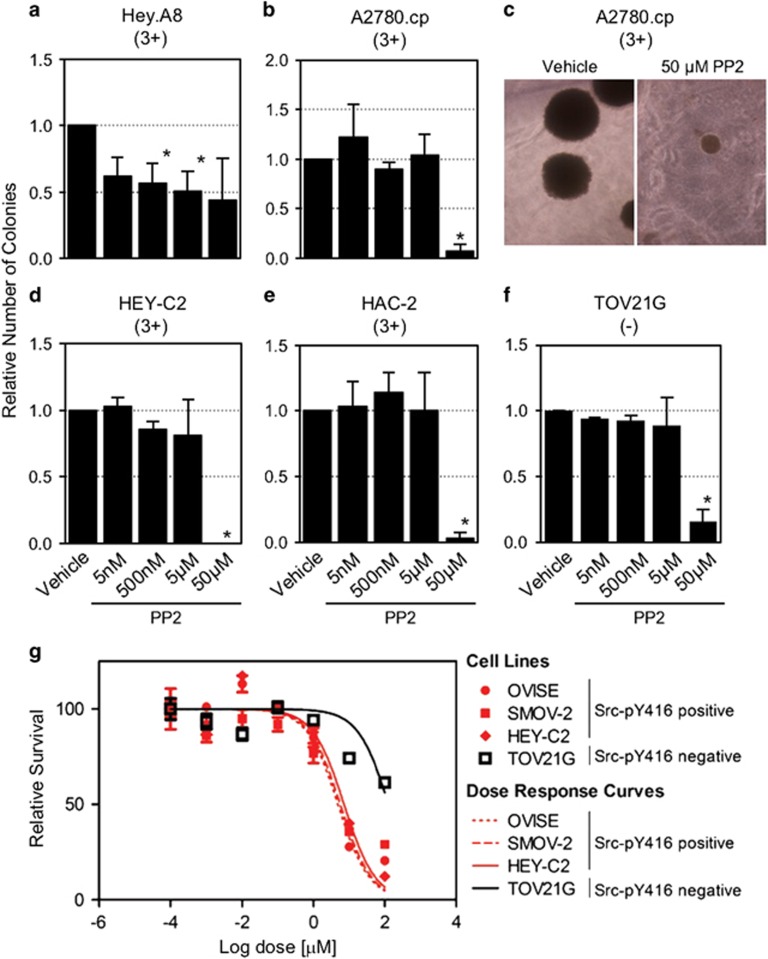
*In vitro* inhibition of Src signaling in anchorage-independent and anchorage-dependent growth assays. (**a–e**) Relative number of colonies in (**a**) Hey.A8, (**b**, **c**) A2780.cp, (**d**) HEY-C2, (**e**) HAC-2 and TOV21G cell lines. (**c**) Representative A2780.cp colonies in cultures treated with vehicle or 50 μm PP2 are shown. Data shown are mean±s.d. of three independent experiments. (**g**) Three Src-pY416-positive and one Src-pY416-negative cell line were assayed for anchorage-dependent growth when treated with PP2. Cells were cultured in the presence of PP2 for 7 days.

We used 4-amino-5-(4-chlorophenyl)-7-(dimethylethyl)pyrazolo[3,4-*d*]pyrimidine (PP2) to inhibit Src in OC cell lines expressing Src-pY416. Addition of PP2 to the cell culture medium inhibits expression of Src-pY416 in OC cells in a dose-dependent manner (Supplementary Figure 1). We first evaluated the effects of PP2 on anchorage-independent growth in four cell lines that form colonies in semi-solid medium—HAC-2 and A2780.cp, which represent models of EAOCs and Hey.A8 and HEY-C2, which represent non-EAOCs. All four showed sensitivity to Src inhibition at a 50 μm PP2 dose (*P<*0.05), while Hey.A8 also exhibited a dose-dependent sensitivity (correlation between dose and colony number, *R*^2^=0.8) (Figures 3a–e). A Src-pY416-negative cell line, TOV21G, also exhibited some sensitivity at 50 μm, suggesting that off-target effects can occur at this dose, although we note that inhibition of colony formation in TOV21G was less than in three of the four Src-pY416-positive lines. In HAC-2, Src inhibition was associated with reduction in colony numbers (Figure 3e) *and* a trend in reduced colony size (Supplementary Figure 2).

Anchorage-dependent growth assays were also performed to test the sensitivity of OC cells to PP2 treatment when cultured on plastic in the presence of PP2 for 7 days. Cell lines positive for Src-pY416 (OVISE, SMOV-2 and HEY-C2) were more sensitive than the Src-pY416-negative TOV21G line (Figure 3g and Supplementary Table 4).

### Inhibiting Src signaling in 3D models of OC

**Figure 4:**
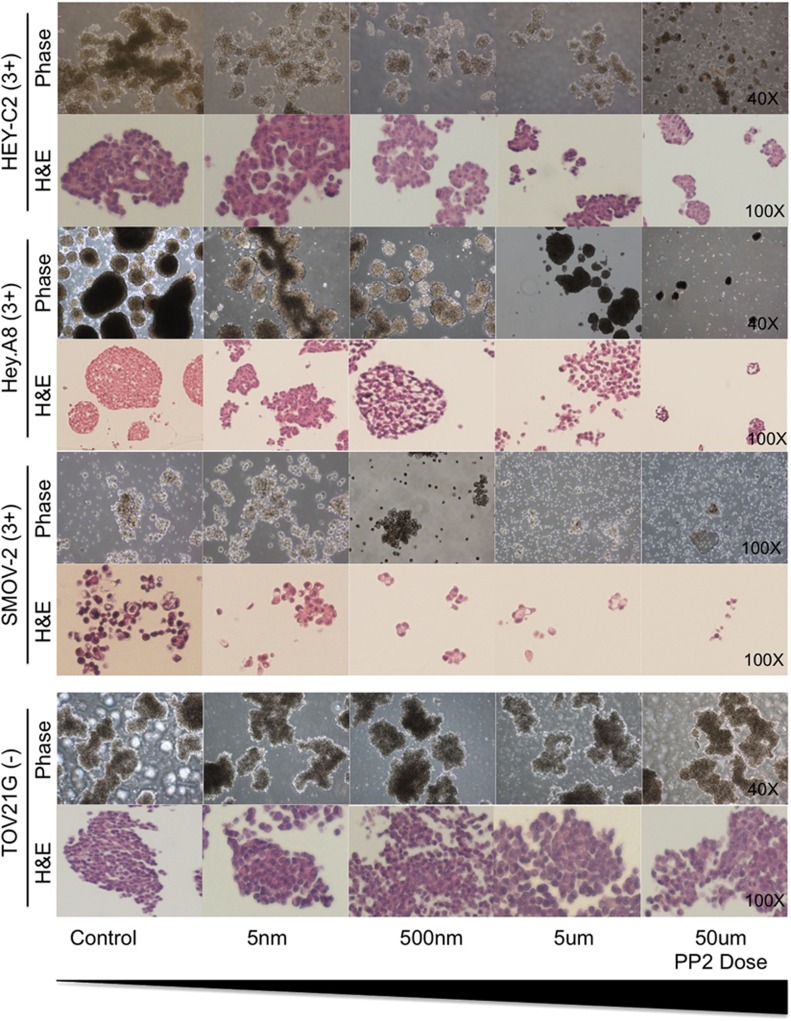
Inhibition of Src signaling in 3D OC models. Three Src-pY416-positive and one Src-pY416-negative cell lines were cultured as 3D models in the presence of various doses of PP2. Cultured were fixed after 7 days of exposure. Phase images and H&E sections enable qualitative assessment of the effects of PP2.

*In vitro* 3D multicellular aggregates (MCAs) mimic the OC aggregates found in the ascitic fluid of OC patients and so we tested whether inhibiting Src can impair the formation of 3D MCAs in Src-pY416-positive and -negative cell lines. Cell line models expressing high levels of active Src were Hey.A8 and HEY-C2 (high-grade OC models but subtype unknown), and SMOV-2 (clear cell OC) (Supplementary Tables 2 and 3). TOV21G was included as a clear cell model that does not express Src-pY416. Cell lines were established as MCAs in the presence or absence of PP2, and cultured for 7 days. In Src-pY416-positive cell lines (Hey.A8, HEY-C2 and SMOV-2), inhibition of Src induced a dose-dependent qualitative reduction in MCA size and number (Figure 4). MCAs were diffuse in PP2-treated Hey.A8 and SMOV-2 cells, indicating cell–cell contacts are disrupted when Src signaling is perturbed, while MCA formation was unaffected in Src-negative TOV21G cells (Figure 4).

## Discussion

Targeted therapies that exploit specific pathways active in a patients' tumor have significantly improved patient outcomes for many cancers. Therapies such as Trastuzumab for HER2-positive breast cancer, and Imatinib for leukemias driven by the BCR-ABL fusion are now widely used in clinical practice. There are currently no targeted therapies routinely used for the treatment of OC, with the exception of olaparib (a PARP1 inhibitor), which recently been approved specifically for the small subset of pre-treated OCs caused by germline mutations in *BRCA1* and *BRCA2*. Novel targeted therapies are needed for non-*BRCA1/2*-associated OCs and particularly for the non-serous histological subtypes, in which *BRCA1/2* mutations are rare. We previously reported Src activation in endometriosis epithelial cells in *in vitro* models of endometriosis and in endometriosis tissues.^14^ It follows therefore that in the current study Src-pY416 positivity is also common in EAOCs. These data suggest a role for Src activation in the development of EAOCs. Src activation has not yet been studied in EAOC but has been studied in depth in mucinous OC, where inhibition of Src reduces cell viability, sensitizes cells to platinum chemotherapy and inhibits cell growth *in vivo*.^12, 13^ Our data suggest that patients with EAOCs could benefit from therapeutic targeting of Src, in particular clear cell OCs that most frequently exhibit Src activation, but also perhaps a subset of serous OCs, one-third of which expresses active Src. Although endometrioid OCs are associated with endometriosis,^7, 9, 10^ activation of Src was not strongly associated with this subtype, suggesting that activation of this pathway may be more commonly involved in the progression of endometriosis to tumors of clear cell histology. Further *in vitro* and *in vivo* modeling will be required to investigate this hypothesis.

To examine the effects of inhibiting Src, we selected *in vitro* assays that aim to mimic the progression of OC *in vivo.* Anchorage-independent growth assays, which mimic dissemination of single tumor cells, showed that treatment of Src-pY416-positive cell lines with a Src inhibitor significantly impeded colony seeding and/or proliferation. In 3D cultures, which are similar to tumor cell aggregates found in ascitic fluid, high-dose Src (50 μm) inhibition impaired or even abolished MCA formation. Qualitative assessment of MCAs suggests that cell–cell adhesion as well as cell survival was impaired by inhibition of Src. This is consistent with the known functions of Src, which operates upstream of many pathways involved in these cellular processes. MCAs in ascitic fluid are responsible for the widespread dissemination of disease throughout the peritoneal cavity, which results in high tumor burden for patients with advanced disease, making treatment of OC particularly challenging. Our data suggest that targeting the Src pathway could be a way to impede the spread of advanced OC, though *in vivo* pre-clinical testing will be required to test the efficacy of such an approach.

In this study, we used PP2 to inhibit Src signaling, as PP2 can inhibit Src activity *in vitro* and impair the growth of colon, ovarian, liver and breast cancer cells *in vitro* and/or *in vivo*.^2, 16, 17^ However, one caveat of using PP2 is that it can also have activity against other SFK members, and we also observed some off-target effects at high doses.^18^ Our approach is consistent with recent clinical trials in which SFK rather than Src-specific inhibitors have been favored, including dasatinib (BMS354825) and saracatinib (AZD0530),^19, 20, 21^ perhaps because in some instances activity against other SFK members may be advantageous.

Cell lines negative for Src-pY416 were not sensitive to PP2 in MCA assays, reinforcing the idea that targeted therapies are of little or no benefit in the absence of pathway activation. Clinical trials targeting Src in OC have not found an improvement in patient outcome; but the OC trials reported to date did not select patients based on Src expression.^19, 20, 21^ Future trials involving only patients with tumors exhibiting active Src signaling will be required to fully evaluate whether inhibiting Src can offer a survival benefit for OC patients with Src-driven cancers. Src activity can be reliably assayed using immunohistochemistry, which could be performed rapidly on tumor biopsies to identify the patients most likely to benefit from Src-targeted therapy. Preliminary data from other tumor types support this approach: in a phase I study exploring the use of dasatinib for chemo-naive metastatic colorectal cancer, 75% of tumors expressing active Src responded to dasatinib, whereas tumors without active Src showed no response.^22^ The timing of SFK inhibition also appears to be important, as data from trials for castration-resistant metastatic prostate cancer suggest that dasatinib may impair bone metastasis in patients who have received no prior chemotherapy.^23, 24^ It appears unlikely that SFK inhibitors will be effective as single agent treatment for recurrent disease;^21^ but data from *in vitro* and *in vivo* models as well as some promising early results from a phase I trial suggest that the combination of a dasatinib plus chemotherapy could improve outcomes for OC patients.^20, 25^

In summary, the current study represents, to our knowledge, the largest study to evaluate Src-pY416 protein expression in OCs of all the major histologies, and the first to specifically implicate Src in the development of EAOCs. These results support the continued investigation of Src and SFK inhibitors as novel therapeutic agents for OC.

## Materials and methods

### Institutional review board approval

All patient samples used in this study were collected with informed patient consent and all protocols were performed with approval from the institutional review boards at the University of Southern California, University College London or Oregon Health and Science University.

### Tissue microarray staining and scoring

The tumor tissue microarrays (TMAs) used in this study have been previously described.^26, 27, 28, 29^ The patient characteristics of the population can be found in Supplementary Table 2, we selected patients with epithelial-type ovarian tumors for inclusion on the TMAs. To stain for phospho-Src (Tyr416), 4 μm sections were freshly cut and then deparaffinized with xylene. Sections were washed with 100% ethanol and incubated with 3% H_2_O_2_ for 10 min. A serum-free protein block (Dakocytomation, Carpinteria, CA, USA) was applied for 30 min before sections were treated with an EDTA buffer saline solution, microwaved (20 min) and then incubated with an anti-phospho Src (Tyr416) monoclonal antibody (Cell Signaling Technologies, Danvers, MA, USA, 1:100 dilution) for 1 h at room temperature. The diaminobenzidine complex was used as a chromagen. Two pathologists (PMF and EP) scored staining intensity and percentage of expressing cells in each sample.

### TMA data analysis

Tabulated data were analyzed in SAS version 9.2 (SAS, Cary, NC, USA) using simple linear regression. For survival analyses, Kaplein–Meier survival estimates with log-rank testing were determined using SPSS (IBM, Armonk, NY, USA). *P*-values less than 0.05 was considered as statistically significant.

### Cell culture

Cell cultures were maintained following standard protocols. Cell culture media used are listed in Supplementary Table 5. Short tandem repeats were profiled to authenticate all cell lines used in the *in vitro* assays and all lines were confirmed to be free of *Mycoplasma* infection.

### 3D culture of epithelial OC cell lines

For analysis of Src activation in OC cell lines, the cell line TMA from Lee *et al.*^15^ was stained for phospho-Src, and we generated a second cell line TMA containing additional 38 cell lines. A list of all cell lines included in this study, and culture media used for each, can be found in Supplementary Table 3. The establishment, fixation and TMA generation of 3D cultures of epithelial OC cell lines was performed as previously described.^15^ Cell culture TMAs were stained and scored for Src-pY416 positivity as described above for primary tumors. To grow 3D spheroids, cell culture dishes were coated twice with 1.5% polyhydroxoethylamethacrylate (polyHEMA) dissolved in 95% ethanol. Cells were trypsinized, resuspended in appropriate growth media and inoculated onto dry polyHEMA-coated plates to induce spheroid formation. PP2 was added to the cultures as required and spheroids were refed every 3–4 days. 3D cultures were harvested after 14 days by centrifugation at 200 *g* for 5 min to sediment the spheroids. Spheroids were washed with phosphate-buffered saline (PBS) and fixed in neutral buffered formalin (VWR, Radnor, PA, USA) for 30 min before processing into paraffin. Images of spheroids were obtained using a Nikon Eclipse Ts100 microscope (Nikon, Tokyo, Japan) and NIS Elements D 3.2 software (Nikon).

### Immunofluorescent staining and western blot analysis

In all, 50 000 SMOV-2 and Hey.A8 cells were plated onto glass coverslips in 6-well plates. After 24 h, PP2 was added, and the cell incubated for a further 48 h. Cells were then fixed with 4% paraformaldehyde (Sigma, St Louis, MO, USA) in PBS and permeabilized in 0.5% Triton-X in PBS. Cells were blocked in DMEM containing 10% fetal bovine serum (VWR) before adding an anti-phospho Src (Tyr416) monoclonal antibody (Cell Signaling Technologies, 1:100 dilution, catalog number 6943) for 1 h. Coverslips were washed before adding an anti-Rabbit Alexa Fluor coupled secondary antibody (Thermo Fisher Scientific, Waltham, MA, USA, 1:250 dilution). Coverslips were washed, stained with Hoechst (Life Technologies, 1:1000), rewashed and mounted using Mowiol (Calbiochem, Billerica, MA, USA). Stained cells were imaged using a Zeiss Axio Imager Z1 fluorescent microscope (Carl Zeiss AG, Oberkochen, Germany). For western blot analyses, cells were treated with PP2 for 48 h, lysed with RIPA buffer (Sigma) containing protease and phosphatase inhibitor cocktails (Roche, Basel, Switzerland). Insoluble proteins were cleared by centrifugation and protein concentrations determined using a BCA Protein Assay (Thermo Fisher Scientific). In all, 30 μg protein was loaded onto a gradient gel, and transfers performed by semi-dry blotting. Membranes were blocked in Odyssey blocking buffer and incubated with primary antibodies. We used the same anti-phospho Src (Tyr416) monoclonal antibody as above, plus a non-phospho Src (Tyr416) (Cell Signaling Technologies, catalog number 2102), both used at a 1:1000 dilution and incubated overnight. Infra-red coupled secondary antibodies were applied for 1 h (IRDye 680 and 800, Licor Biosciences, Lincoln, NE, USA) and the membranes scanned using the Odyssey Imaging System (Licor Biosciences). Quantification was performed using Image Studio Lite software (Version 5, Licor Biosciences), with background normalization. Immunostaining and blots were performed at least twice. Raw quantification data for western blots after PP2 treatment can be found in Supplementary Table 6.

### Anchorage-independent growth assays

Assays were performed with PP2 (Tocris Bioscience, Pittsburgh, PA, USA) at doses of 50 μm, 5 μm, 500 nm and 5 nm. Control cells received vehicle (0.1% DMSO, Sigma) alone. Anchorage-independent growth assays were performed in 6-well plates by plating 2 × 10^4^ cells per well in 3.3% Noble agar (Sigma) top layer on top of a 6% agar base layer. Assays were incubated for 4 weeks and then stained with 1% p-iodonitrotetrazolium violet (Sigma) dissolved in methanol, to fix and stain cells to enable enumeration of the colonies. Colonies were counted and imaged using a Nikon Eclipse TS100 microscope and NIS Element D 3.2 software. Two-tailed paired Student's *T*-tests were used to test for phenotypic differences in PP2-treated and untreated cells. Assays were performed three times, with triplicate wells plated for each dose of drug.

### Anchorage-dependent growth assays

In all, 1500 HEY-C2, 3000 SMOV-2, 5000 OVISE or 1500 TOV21G cells were plated per well, in 24-well plates. After 24 h, seven doses of PP2 were added, and the cells were incubated for 7 days. Control cells received vehicle (0.1% DMSO) alone. Media (containing PP2 or vehicle) was replenished on day 3 or 4. After 7 days, 100 μl of 3-(4,5-dimethylthiazol-2-yl)-2,5-diphenyltetrazolium bromide (MTT) solution (10 mg/ml in PBS) was applied to each well and incubated for 2 h. Cells were washed with PBS and lysed in DMSO (Sigma). Absorbance was read using a Mikrowin platereader (Medical Scientific, Muenster, Germany). Data were analyzed in GraphPad Prism (Graphpad Software, Inc, La Jolla, CA, USA). Treatment doses were log transformed, and the non-linear curve of best fit was calculated.

### Treatment of 3D cultures with PP2

For PP2 treatment of 3D cultures, cells were grown in 3D as described above and, at the time of seeding, culture media supplemented with PP2 at the specified doses, or vehicle (0.1% DMSO). Cells were treated for 7 days before harvesting. Samples were processed in paraffin blocks and hematoxylin and eosin-stained sections made by the USC Pathology Core facility.

## Figures and Tables

**Table 1:** Analysis of Src-pY416 in primary epithelial ovarian cancer specimens

	*Negative*, N *(%)*	*Positive*, N *(%)*	P*-value*
*Stage*			
Stage 1	49 (68)	23 (32)	
Stage 2	21 (66)	11 (34)	
Stage 3	144 (61)	91 (39)	
Stage 4	28 (74)	10 (26)	0.415
Stage 1/2	70 (67)	34 (33)	
Stage 3/4	172 (63)	101 (37)	0.510
			
*Grade*			
FIGO1/2	26 (67)	13 (33)	
FIGO3	217 (64)	124 (36)	0.844
			
*Histology*			
Serous	166 (66)	86 (34)	
Clear cell	17 (41)	24 (59)	
Mucinous	17 (81)	4 (19)	
Endometrioid	22 (61)	14 (39)	
Mixed	10 (63)	6 (37)	0.017
EAOC^a^	39 (51)	38 (49)	
Non-EAOC	183 (67)	90 (33)	0.011
Serous	166 (66)	86 (34)	
Non-Serous	56 (57)	42 (43)	0.139
			
*Optimal debulking*			
No	102 (63)	61 (37)	
Yes	138 (65)	74 (35)	0.6929
			
*Recurrence*			
No	73 (66)	38 (34)	
Yes	87 (63)	64 (37)	0.2006

Abbreviation: EAOC, endometriosis-associated ovarian cancer.

381 specimens were analyzed by immunohistochemical staining for phospho-Src (Tyr416). *P*-values represent results from simple linear regression. Percentages denote the percent of positive and negative tumors within each group, which is indicated by the row name.

a Clear cell and endometrioid compared with serous and mucinous; mixed tumors were excluded.
