# Supplementary material for: Targeting Src in endometriosis-associated ovarian cancer
Source: Oncogenesis. 2016 Aug 15;5(8):e251–. doi: 10.1038/oncsis.2016.54 (PMC5007828; doi:10.1038/oncsis.2016.54)
Supplement: Supplementary Information [file oncsis201654x1.docx]

**Src Activation in Endometriosis-Associated Ovarian Cancer**

**Supplementary Tables 1-5**

**Supplementary Figures 1&2**

**Supplementary Table 1. Characteristics of patient population used for analysis of Src activation in primary tumours.** ‘Other’ histologies includes transitional cell carcinoma, carcinosarcoma, and undifferentiated tumours.

|  | **N (%)** |
| --- | --- |
| **Stage** |  |
| Stage 1 | 72 (18.9) |
| Stage 2 | 32 (8.4) |
| Stage 3 | 235 (61.7) |
| Stage 4 | 38 (10.0) |
| Not known | 4 (1.1) |
|  |  |
| **Grade** |  |
| FIGO1/2 | 39 (10/2) |
| FIGO3 | 341 (89.5) |
| Not known | 1 (0.3) |
|  |  |
| **Histology** |  |
| Serous | 252 (66.1) |
| Clear cell | 41 (10.8) |
| Mucinous | 21 (5.5) |
| Endometrioid | 36 (9.4) |
| Mixed | 16 (4.2) |
| Other | 12 (3.2) |
| Not specified | 3 (0.8) |
|  |  |
| **Optimal Debulking** |  |
| No | 163 (42.8) |
| Yes | 138 (55.6) |
| Not known | 6 (1.6) |
|  |  |
| **Recurrence** |  |
| No | 111 (29.1) |
| Yes | 251 (65.9) |
| Not known | 19 (5.0) |

**Supplementary Table 2. Cell lines histologies in 2D and 3D culture.** Cell lines are sorted by primary tumor histology. CCOC, clear cell ovarian cancer; HG, high grade; HGSOC, high grade serous ovarian cancer; MOC, mucinous ovarian cancer; HG, no growth; NS, not specified; SOC, serous ovarian cancer; TER, teratocarcinoma. * some mucine present. Data for an additional 31 cell lines have been published previously (1). Cell lines where the original reported histology and 3D histology are different are highlighted in grey.

| **Cell Line** | **Tumor Histology** | **2D Histology** | **3D Histology** | **Reference** |
| --- | --- | --- | --- | --- |
| HAC2 | CCOC | HG | HG (papillary) |  |
| HCH-1 | CCOC | HG | CCOC | (2) |
| JHOC-5 | CCOC | HG | CCOC | (3) |
| JHOC-7 | CCOC | CCOC | CCOC |  |
| JHOC-9 | CCOC | CCOC | CCOC |  |
| KK | CCOC | HGSOC | HGSOC | (4) |
| KOC-7c | CCOC | HG | CCOC |  |
| OV207 | CCOC | CCOC | HG / CCOC | (5) |
| OVAS | CCOC | HG | HG / necrotic | (6) |
| OVISE | CCOC | HG | CCOC | (7, 8) |
| OVMANA | CCOC | HG | CCOC | (8) |
| OVSAYO | CCOC | HG | NG | (8) |
| OVTOKO | CCOC | HG | CCOC | (7, 8) |
| RMG-I | CCOC | HG | CCOC | (9) |
| RMG-II | CCOC | HG | CCOC | (10) |
| SMOV-2 | CCOC | HG | CCOC | (11) |
| TU-OC-1 | CCOC | HG | CCOC | (12) |
| OV167 | HGSOC | HG | HG | (5) |
| OV177 | HGSOC | HG | HGSOC | (5) |
| OVKATE | HGSOC | HG | CCOC | (8) |
| OVSAHO | HGSOC | HC | NG | (8) |
| SHIN-3 | HGSOC | HG | HGSOC | (13) |
| EFO-21 | SOC | HG | HG | (14) |
| PEO6 | SOC | HG | CCOC | (15) |
| MCAS | MOC | HG | MOC | (16) |
| TU-OM-1 | MOC | HG | NG |  |
| COLO-704 | NS | HG | HG |  |
| DOV-13 | NS | HG | Necrotic |  |
| HEY-C2 | NS | HG | CCOC |  |
| KURAMOCHI | NS | HG | HGSOC | (17) |
| OAW28 | NS | HG | NG | (18) |
| OVCA432 | NS | HG* | HG* |  |
| OVCAR8 | NS | HG | HG |  |
| TYK-nu | NS | HG | HG | (19) |
| PA-1 | TER | HG | HG |  |

**Supplementary Table 3. Src expression in epithelial ovarian cancer cell lines, cultured in 2D and 3D.** Expression of p-Src (Tyr416) was scored as negative (0), weak (1), moderate (2) or strong (3). NG, no growth in 3D; NA, not available (insufficient material for scoring). Positive cell lines are highlighted in bold font.

| **Cell line** | **2D** | **3D** |
| --- | --- | --- |
| 1847 | 0 | 0 |
| 1847.AD | 0 | 0 |
| A2780 | 0 | 0 |
| **A2780.CP** | **2** | **2** |
| CaOV3 | 0 | 0 |
| **COLO-704** | **2** | **1** |
| COV318 | 0 | 0 |
| COV434 | 0 | 0 |
| **COV644** | **2** | **2** |
| **DOV 13** | **3** | **0** |
| **EFO 21** | **2** | **0** |
| EFO27 | 0 | 0 |
| ES-2 | 0 | 0 |
| FUOV1 | 0 | 0 |
| **HAC-2** | **3** | **3** |
| **HCH-1** | **1** | **0** |
| **Hey.A8** | **3** | **3** |
| **HEYC2** | **3** | **3** |
| IGROV1 | 0 | 0 |
| **JAMA-2** | **3** | **3** |
| **JHOC5** | **1** | **0** |
| **JHOC7** | **3** | **1** |
| **JHOC9** | **2** | **0** |
| **KK** | **1** | **1** |
| **KOC-7c** | **0** | **1** |
| **KURAMOCHI** | **1** | **1** |
| LK2 | 0 | 0 |
| **MCAS** | **NA** | **2** |
| OAW28 | NA | NA |
| OAW42 | 0 | 0 |
| OC316 | 0 | 0 |
| **OV 167** | **2** | **2** |
| **OV 177** | **1** | **1** |
| **OV 207** | **3** | **1** |
| **OV-MZ-15** | **1** | **NA** |
| **OVAS** | **2** | **2** |
| OVCA429 | 0 | NG |
| OVCA432 | NA | 0 |
| OVCA433 | 0 | NG |
| **OVCAR 8** | **0** | **3** |
| OVCAR10 | 0 | 0 |
| OVCAR3 | 0 | 0 |
| OVCAR5 | 0 | 0 |
| **OVISE** | **3** | **3** |
| **OVKATE** | **3** | **NA** |
| **OVMANA** | **2** | **0** |
| OVSAHO | 0 | 0 |
| **OVSAYO** | **3** | **NA** |
| OVTOKO | 0 | NA |
| PA-1 | 0 | 0 |
| **PEO14** | **2** | **NA** |
| **PEO6** | **3** | **NA** |
| PXN94 | 0 | 0 |
| **RMG-I** | **2** | **NA** |
| RMG-II | NA | NA |
| SHIN-3 | 0 | 0 |
| SKOV3 | 0 | 0 |
| SKOV3.ip | 0 | 0 |
| **SMOV2** | **3** | **3** |
| TOV112D | 0 | 0 |
| TOV21G | 0 | 0 |
| **TU-OC1** | **1** | **0** |
| **TU-OM-1** | **3** | **NA** |
| **TYK-nu** | **3** | **3** |
| UWB1.289 | 0 | 0 |
| UWB1.289 + BRCA1 | 0 | 0 |

**Supplementary Table 4. IC50 values for PP2 treated ovarian cancer cell lines.**

| **Cell Line** | **Src-pY416 Status** | **IC50 (95% CI)** | |
| --- | --- | --- | --- |
| OVISE | Positive | 4.85 (3.19-7.38) | |
| SMOV-2 | Positive | 5.62 (3.04-10.38) | |
| HEY-C2 | Positive | 7.18 (4.85-10.63) | |
| TOV21G | Negative | 128.3 (77.45-212.5) | |

**Supplementary Table 5. Cell lines and culture media used.**  CR UK, Cancer Research UK, FBS, foetal bovine serum; NEAA, non-essential amino acids

| **Cell Line** | **Culture Media** | **Source** |
| --- | --- | --- |
| 1847 | RPMI 1640 +10% FBS + L-Glu | CR UK |
| 1847 AD | RPMI 1640 +10% FBS + L-Glu | CR UK |
| A2780 | RPMI 1640 +10% FBS + L-Glu | Dr G Mills |
| A2780CP | DMEM:F12 + 10% FBS + L-Glu | Dr B Tsang |
| CaOV3 | DMEM + 10% FBS + 1X NEAA | CR UK |
| COLO-704 | RPMI 1640 +10% FBS + L-Glu + 1X NEAA + 100ug/ml insulin | Dr M Press |
| COV644 | DMEM +10% FBS+Lglu+Lasparagine | Dr P Schrier |
| COV318 | DMEM +10% FBS+Lglu+Lasparagine | Dr P Schrier |
| COV434 | DMEM +10% FBS+Lglu+Lasparagine | Dr P Schrier |
| DOV13 | EMEM + 15% FBS + L-Glu | Dr M Press |
| EFO 21 | RPMI 1640 +10% FBS + L-Glu + 1X NEAA + 100ug/ml insulin | Dr M Press |
| EFO 27 | RPMI 1640 + 20% FBS + L-Glu + NEAA + Na Pyr | Dr G Mills |
| EN-TRL-71T | M199:MCDB105 + 15% FBS+ L-Glu | Created at UCL |
| ES-2 | RPMI 1640 +10% FBS + L-Glu | CR UK |
| FUOV1 | DMEM:F12 + 10% FBS + L-Glu | Dr G Mills |
| HAC-2 | RPMI 1640 +10% FBS + L-Glu | Dr H Itamochi |
| HCH-1 | DMEM:F12 + 10% FBS + L-Glu | Dr H Itamochi |
| Hey.A8 | RPMI 1640 +10% FBS + L-Glu | Dr G Mills |
| HEYC2 | DMEM:F12 + 10% FBS + L-Glu | Dr M Press |
| IGROV | DMEM + 10%FBS + L-Glu | Dr G Mills |
| INTOV-2 | RPMI 1640+ 10% FBS+ LGlu+ 50uL β ME +1% Na Pyr | Dr D Mezzanzanica |
| JAMA-2 | RPMI 1640 +10% FBS + L-Glu | CR UK |
| KK | RPMI 1640 +10% FBS + L-Glu | Dr H Itamochi |
| KOC-7c | RPMI 1640 +10% FBS + L-Glu | Dr H Itamochi |
| KURAMOCHI | RPMI 1640 +10% FBS + L-Glu + 1X NEAA + 100ug/ml insulin | Dr M Press |
| LK2 | RPMI 1640 +10% FBS + L-Glu | CR UK |
| MCAS | EMEM + 15% FBS + L-Glu | Dr M Press |
| MPSC1 | RPMI 1640 +10% FBS + L-Glu | Dr G Mills |
| OAW 42 | DMEM + 10% FBS + Na Pyr + 20ug/ml Insulin + L-Glu | Dr G Mills |
| OAW28 | DMEM + 10% FBS + L-Glu + 100ug/ml insulin | Dr M Press |
| OC316 | RPMI 1640 +10% FBS + L-Glu | Dr G Mills |
| OV167 | Alpha MEM + 10% FBS + L-Glu | Dr M Press |
| OV177 | Alpha MEM + 10% FBS + L-Glu | Dr M Press |
| OV2008 | RPMI 1640 +10% FBS + L-Glu | Dr B Tsang |
| OV2008 C13* | RPMI 1640 +10% FBS + L-Glu | Dr B Tsang |
| OV207 | Alpha MEM + 10% FBS + L-Glu | Dr M Press |
| OVAS | RPMI 1640 +10% FBS + L-Glu | Dr H Itamochi |
| OVCA 420 | Alpha MEM + 10% FBS + L-Glu + 1X NEAA + 1X Na-Pyr | Dr M Press |
| OVCA 429 | MEM EAGLE + 10% FBS + NEAA + L-Glut + Na Pyr | Dr G Mills |
| OVCA 432 | EMEM + 15% FBS + L-Glu | Dr M Press |
| OVCA 433 | MEM EAGLE + 10% FBS + L-Glu | Dr G Mills |
| OVCAR 3 | RPMI 1640 +10% FBS + L-Glu | CR UK |
| OVCAR 5 | RPMI 1640 +10% FBS + L-Glu | Dr G Mills |
| OVCAR 8 | RPMI 1640 +10% FBS + L-Glu | CR UK |
| OVISE | RPMI 1640 +10% FBS + L-Glu | Dr H Itamochi |
| OVKATE | RPMI 1640 +10% FBS + L-Glu | Dr M Press |
| OVMANA | RPMI 1640 +10% FBS + L-Glu | Dr H Itamochi |
| OVP1 | RPMI 1640 +10% FBS + L-Glu | Dr G Natali |
| OVSAHO | RPMI 1640 +10% FBS + L-Glu | Dr M Press |
| OVSAYO | RPMI 1640 +10% FBS + L-Glu | Dr H Itamochi |
| OVTOKO | RPMI 1640 +10% FBS + L-Glu | Dr H Itamochi |
| PEA2 | RPMI 1640 +10% FBS + L-Glu + 1X NEAA + 100ug/ml insulin | Dr M Press |
| PEO6 | DMEM:F12 + 10% FBS + L-Glu | Dr M Press |
| PXN94 | RPMI 1640 +10% FBS + L-Glu | CR UK |
| RMG-I | RPMI 1640 +10% FBS + L-Glu | Dr H Itamochi |
| RMG-II | RPMI 1640 +10% FBS + L-Glu | Dr H Itamochi |
| SHIN-3 | DMEM:F12 + 10% FBS + L-Glu | Dr H Itamochi |
| SKOV 3 IP | RPMI 1640 +10% FBS + L-Glu | Dr G Milles |
| SKOV3 | RPMI 1640 +10% FBS + L-Glu | ATCC |
| SMOV-2 | RPMI 1640 +10% FBS + L-Glu | Dr H Itamochi |
| TOV112D | M199:MCDB105 + 15% FBS+ L-Glu | ATCC |
| TOV21G | M199:MCDB105 + 15% FBS+ L-Glu | ATCC |
| TU-OC1 | RPMI 1640 +10% FBS + L-Glu | Dr H Itamochi |
| TU-OM-1 | DMEM:F12 + 10% FBS + L-Glu | Dr H Itamochi |
| TYK-nu | EMEM + 15% FBS + L-Glu | Dr M Press |
| UWB1.289 | 1:1 MEGM:RPMI, 3% FBS | ATCC |
| UWB1.289 + BRCA1 | 1:1 MEGM:RPMI, 3% FBS, 200ug/ml G418 | ATCC |

**Supplementary Table 6. Raw data for Western blots measuring phosphorylated and non- phosphorylated Src expression following PP2 treatment.**  Background normalization was performed prior to calculating the ratio of phosphorylated to non- phosphorylated Src.

| **BLOT 1** |  |  |  |  |  |
| --- | --- | --- | --- | --- | --- |
|  | **tx** | **p src 416** | **non p src** | **ratio** | **Relative to DMSO** |
| **HEYA8** | **DMSO** | 327 | 364 | 0.90 | 1.00 |
|  | **50nM** | 299 | 361 | 0.83 | 0.92 |
|  | **500nM** | 169 | 454 | 0.37 | 0.41 |
|  | **5uM** | 156 | 329 | 0.47 | 0.53 |
|  | **50uM** | 42 | 121 | 0.35 | 0.39 |
| **SMOV2** | **DMSO** | 404 | 413.5 | 0.98 | 1.00 |
|  | **50nM** | 349 | 350 | 1.00 | 1.02 |
|  | **500nM** | 126 | 284 | 0.44 | 0.45 |
|  | **5uM** | 35 | 101 | 0.35 | 0.35 |
|  |  |  |  |  |  |
|  |  |  |  |  |  |
| **BLOT 2** |  |  |  |  |  |
|  | **tx** | **p src 416** | **non p src** | **ratio** | **Relative to DMSO** |
| **HEYA8** | **DMSO** | 10500 | 11600 | 0.91 | 1.00 |
|  | **50nM** | 9050 | 10900 | 0.83 | 0.92 |
|  | **500nM** | 5710 | 14300 | 0.40 | 0.44 |
|  | **5uM** | 3220 | 16200 | 0.20 | 0.22 |
|  | **50uM** | 192 | 6910 | 0.03 | 0.03 |
| **SMOV2** | **DMSO** | 28100 | 31400 | 0.89 | 1.00 |
|  | **50nM** | 13500 | 30600 | 0.44 | 0.49 |
|  | **500nM** | 6100 | 21500 | 0.28 | 0.32 |
|  | **5uM** | 1530 | 9750 | 0.16 | 0.18 |
|  |  |  |  |  |  |
|  |  |  |  |  |  |
| **COMBINED** | |  |  |  |  |
|  | **tx** | **Avg** | **SD** |  |  |
| **Hey.A8** | **Vehicle** | 1.00 | 0.0 |  |  |
|  | **50nM** | 0.92 | 0.0 |  |  |
|  | **500nM** | 0.43 | 0.0 |  |  |
|  | **5uM** | 0.37 | 0.2 |  |  |
|  | **50uM** | 0.21 | 0.3 |  |  |
| **SMOV2** | **Vehicle** | 1.00 | 0.0 |  |  |
|  | **50nM** | 0.76 | 0.4 |  |  |
|  | **500nM** | 0.39 | 0.1 |  |  |
|  | **5uM** | 0.27 | 0.1 |  |  |

**Supplementary Figure 1. PP2 inhibits Src-p416 expression in ovarian cancer cells.** Immunofluorescent staining in (a) SMOV-2 and (b) Hey.A8, 200X magnification, constant exposure for Src-pY416. Active Src is expressed at the plasma membrane, and expression is decreased in the presence of PP2, in a dose-dependent manner. (c) Western blot analysis of phosphorylated and non-phosphorylated Src (for residue tyrosine 416). Bands were quantified and phospho-Y416 Src levels normalized to non-phospho-Y416 levels. Values shown indicate normalized Src-pY416 levels relative to DMSO vehicle (V) treated cells. For SMOV-2 extensive cell death at the 50 μM dose precluded Western blot analyses. (d) Quantification of Western blots measuring relative Src-pY416 expression. Mean ± standard deviation of two independent blots. Raw data can be found in Supplementary Table 6.

**
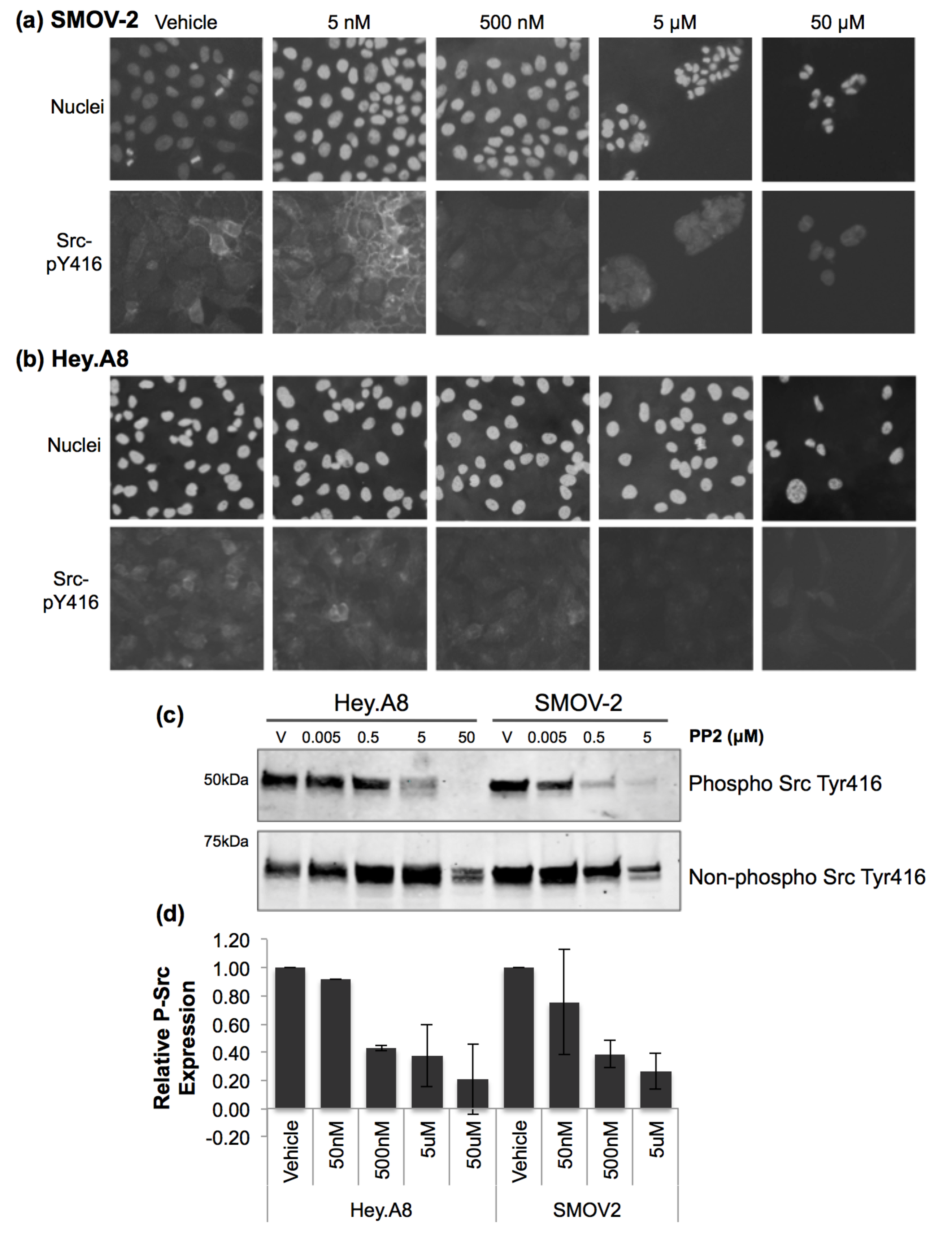
**

**Supplementary Figure 2.** In HAC-2, PP2 treatment was associated with a trend in reduced colony size (* P<0.05).

**
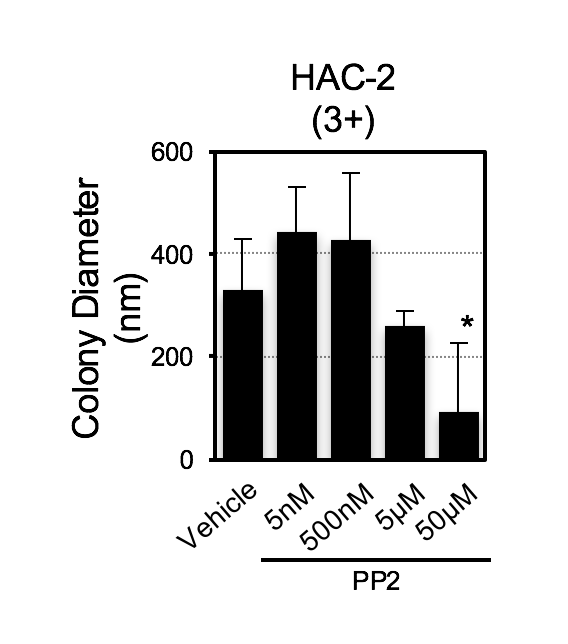
**

**References**

1. Lee JM, Mhawech-Fauceglia P, Lee N, Parsanian LC, Lin YG, Gayther SA, et al. A three-dimensional microenvironment alters protein expression and chemosensitivity of epithelial ovarian cancer cells in vitro. Lab Invest. 2013.

2. T Y, H. K. Establishment and characterization of a cell line (HCH-1) derived from human clear cell adenocarcinoma of the ovary. . Fifty-Eighth Annual Meeting of the Japanese Cancer Association 1999. p. 589A.

3. Yamada K, Tachibana T, Hashimoto H, Suzuki K, Yanagida S, Endoh H, et al. Establishment and characterization of cell lines derived from serous adenocarcinoma (JHOS-2) and clear cell adenocarcinoma (JHOC-5, JHOC-6) of human ovary. Hum Cell. 1999;12(3):131-8.

4. Sasa H, Ishii K, Hirata J, Kikuchi Y, Nagata I, Kawai T, et al. [Establishment and characterization of a CA125-producing human ovarian clear cell carcinoma cell line]. Hum Cell. 1993;6(4):279-86.

5. Conover CA, Hartmann LC, Bradley S, Stalboerger P, Klee GG, Kalli KR, et al. Biological characterization of human epithelial ovarian carcinoma cells in primary culture: the insulin-like growth factor system. Exp Cell Res. 1998;238(2):439-49.

6. T M, H K, T. S. Establishment and characterization of a CA-125 producing cell line (OVAS-21) from a clear cell adenocarcinoma of the ovary. Hum Cell1988. p. 5347.

7. Gorai I, Nakazawa T, Miyagi E, Hirahara F, Nagashima Y, Minaguchi H. Establishment and characterization of two human ovarian clear cell adenocarcinoma lines from metastatic lesions with different properties. Gynecol Oncol. 1995;57(1):33-46.

8. Yanagibashi T, Gorai I, Nakazawa T, Miyagi E, Hirahara F, Kitamura H, et al. Complexity of expression of the intermediate filaments of six new human ovarian carcinoma cell lines: new expression of cytokeratin 20. Br J Cancer. 1997;76(7):829-35.

9. Nozawa S, Tsukazaki K, Sakayori M, Jeng CH, Iizuka R. Establishment of a human ovarian clear cell carcinoma cell line (RMG-I) and its single cell cloning--with special reference to the stem cell of the tumor. Hum Cell. 1988;1(4):426-35.

10. Nozawa S, Yajima M, Sasaki H, Tsukazaki K, Aoki D, Sakayori M, et al. A new CA125-like antigen (CA602) recognized by two monoclonal antibodies against a newly established ovarian clear cell carcinoma cell line (RMG-II). Jpn J Cancer Res. 1991;82(7):854-61.

11. K Y, K H, T I. Establishment and characterization of human ovarian clear cell adenocarcinoma cell line (SMOV-2), and its cytotoxity by anticancer agent. Hum Cell.1999. p. 139-48.

12. Itamochi H, Kato M, Nishimura M, Oumi N, Oishi T, Shimada M, et al. Establishment and characterization of a novel ovarian clear cell adenocarcinoma cell line, TU-OC-1, with a mutation in the PIK3CA gene. Hum Cell. 2013;26(3):121-7.

13. Imai S, Kiyozuka Y, Maeda H, Noda T, Hosick HL. Establishment and characterization of a human ovarian serous cystadenocarcinoma cell line that produces the tumor markers CA-125 and tissue polypeptide antigen. Oncology. 1990;47(2):177-84.

14. Simon WE, Albrecht M, Hänsel M, Dietel M, Hölzel F. Cell lines derived from human ovarian carcinomas: growth stimulation by gonadotropic and steroid hormones. J Natl Cancer Inst. 1983;70(5):839-45.

15. Langdon SP, Lawrie SS, Hay FG, Hawkes MM, McDonald A, Hayward IP, et al. Characterization and properties of nine human ovarian adenocarcinoma cell lines. Cancer Res. 1988;48(21):6166-72.

16. Kidera Y, Yoshimura T, Ohkuma Y, Iwasaka T, Sugimori H. [Establishment and characterization of a cell line derived from mucinous cystadenocarcinoma of human ovary]. Nihon Sanka Fujinka Gakkai Zasshi. 1985;37(9):1820-4.

17. Motoyama T. [Quantitative analysis on in vitro drug sensitivity of cultured human ovarian cancer cell lines (author's transl)]. Nihon Sanka Fujinka Gakkai Zasshi. 1982;34(3):308-14.

18. Hills CA, Kelland LR, Abel G, Siracky J, Wilson AP, Harrap KR. Biological properties of ten human ovarian carcinoma cell lines: calibration in vitro against four platinum complexes. Br J Cancer. 1989;59(4):527-34.

19. Yoshiya N. [Establishment of a cell line from human ovarian cancer (undifferentiated carcinoma of FIGO classification) and analysis of its cell-biological characteristics and sensitivity to anticancer drugs]. Nihon Sanka Fujinka Gakkai Zasshi. 1986;38(10):1747-53.
